# Inhibition of carbonic anhydrases IX/XII by SLC-0111 boosts cisplatin effects in hampering head and neck squamous carcinoma cell growth and invasion

**DOI:** 10.1186/s13046-022-02345-x

**Published:** 2022-04-02

**Authors:** Annachiara Sarnella, Ylenia Ferrara, Luigi Auletta, Sandra Albanese, Laura Cerchia, Vincenzo Alterio, Giuseppina De Simone, Claudiu T. Supuran, Antonella Zannetti

**Affiliations:** 1 Institute of Biostructures and Bioimaging-CNR, Via T. De Amicis, 95, 80145 Naples, Italy; 2 Institute of Experimental Endocrinology and Oncology “Gaetano Salvatore”, CNR, Via S. Pansini 5, 80131 Naples, Italy; 3 NEUROFARBA Department, Sezione di Scienze Farmaceutiche, University of Florence, Via Ugo Schiff, 6, 50019 Sesto Fiorentino, Florence, Italy

**Keywords:** Head and neck squamous carcinomas, Tumor microenvironment, Cisplatin resistance, Carbonic anhydrase isoenzymes IX and XII

## Abstract

**Background:**

Hypoxic tumor microenvironment (TME) contributes to the onset of many aspects of the cancer biology associated to the resistance to conventional therapies. Hypoxia is a common characteristic and negative prognostic factor in the head and neck squamous carcinomas (HNSCC) and is correlated with aggressive and invasive phenotype as well as with failure to chemo- and radio-therapies. The carbonic anhydrase isoenzymes IX and XII (CA IX/XII), regulators of extra and intracellular pH, are overexpressed in TME and are involved in adaptative changes occurring in cancer cells to survive at low O_2_. In this study, we aim to investigate in HNSCC cells and murine models the possibility to target CA IX/XII by the specific inhibitor SLC-0111 to potentiate the effects of cisplatin in hampering cell growth, migration and invasion. Furthermore, we analyzed the signal pathways cooperating in acquisition of a more aggressive phenotype including stemness, epithelial-mesenchymal transition and apoptotic markers.

**Methods:**

The effects of cisplatin, CA IX/XII specific inhibitor SLC-0111, and the combinatorial treatment were tested on proliferation, migration, invasion of HNSCC cells grown in 2D and 3D models. Main signal pathways and the expression of stemness, mesenchymal and apoptotic markers were analyzed by western blotting. Molecular imaging using NIR-Annexin V and NIR-Prosense was performed in HNSCC xenografts to detect tumor growth and metastatic spread.

**Results:**

HNSCC cells grown in 2D and 3D models under hypoxic conditions showed increased levels of CA IX/XII and greater resistance to cisplatin than cells grown under normoxic conditions. The addition of CA IX/XII inhibitor SLC-0111 to cisplatin sensitized HNSCC cells to the chemotherapeutic agent and caused a reduction of proliferation, migration and invasiveness. Furthermore, the combination therapy hampered activation of STAT3, AKT, ERK, and EMT program, whereas it induced apoptosis. In HNSCC xenografts the treatment with cisplatin plus SLC-0111 caused an inhibition of tumor growth and an induction of apoptosis as well as a reduction of metastatic spread at a higher extent than single agents.

**Conclusion:**

Our results highlight the ability of SLC-0111 to sensitize HNSCC to cisplatin by hindering hypoxia-induced signaling network that are shared among mechanisms involved in therapy resistance and metastasis.

**Supplementary Information:**

The online version contains supplementary material available at 10.1186/s13046-022-02345-x.

## Background

The head and neck squamous carcinomas (HNSSC) are placed at the sixth place in the world for incidence among all solid human malignancies. Most of them consists of oral (OSCC) and oropharyngeal carcinomas (OPSCC). At present, the five-years survival for these tumors is less than 50%, despite the gradual implementation of screening programs and primary prevention against the well-known risk factors such as tobacco and alcohol or infections with human papillomavirus (HPV +) in prevalence of sexually-transmitted type 16. Despite the availability of more advanced chemo-radiation and surgical treatments, mortality for HNSSC remained virtually unchanged over the last twenty years. This is largely related to their resistance to radio and chemotherapy, aggravated by the relevant toxicity and side effects, resulting in overall poor quality of life for patients after radical surgical treatments and challenging and complex reconstructive procedures [1]. The first option to treat recurrent and metastatic HNSCC is cisplatin (Cis-Pt) but unfortunately, many patients develop resistance to this drug [2].

It is well-known the crucial role played by microenvironment in promoting aggressiveness of solid tumors in terms of metastases, disease relapse and radio/chemo-resistance [3–5]. A characteristic of advanced cancer is the presence of a hypoxic microenvironment, due to an aberrant vascularization and a poor blood supply, driving a lethal phenotype [6]. Hypoxia impacts on many aspects of the cancer biology including the resistance to conventional therapies and the ability of tumor cells to metastasize [7]. In particular, many findings demonstrated that the hypoxic tumor microenvironment (TME) is the most important driver in the failure of Cis-PT treatment in many aggressive carcinomas [8]. The expression of cell surface pH regulating enzymes such as carbonic anhydrase IX and XII (CA IX/XII) is increased by hypoxic TME. Both CA isoforms contribute to increase extracellular acidosis by catalyzing the reversible hydration of carbon dioxide to bicarbonate and proton thus creating a suitable place where tumor cells acquire characteristics of stemness and the ability to metastasize, resist treatments and escape immunosurveillance [9]. Noteworthy, the expression of these enzymes is very high in many aggressive solid carcinomas and is correlated with poor prognosis and outcome [10]. Therefore, many studies have focused on the development of small-molecules and antibodies to therapeutically target CA IX/XII as single agents or in combination with conventional therapies [10]. Recently, it has been reported the ability of the sulfonamide inhibitor, SLC-0111, in Phase Ib/II clinical trials (NCT02215850), to sensitize cancer cells to different chemotherapeutic treatments [11]. The co-administration of this drug with temozolamide caused a substantial decrease in the growth of glioblastoma patient-derived xenografts as well as a significant reduction of therapy-resistant brain tumor initiating cells [12].

Interestingly, Chafe et al. demonstrated that the inhibition of CA IX with SLC-0111 increased the effect of immuno-checkpoint blockade by reverting the acidic TME in melanoma and breast cancer xenografts [13]. Furthermore, the use of this inhibitor with gemcitabine prolonged survival and increased cell death in KRAS-driven pancreatic ductal adenocarcinoma xenografts [14]. Herein, we show the potential of CA IX/XII inhibitor, SLC-0111, to sensitize HNSCC tumor cells and xenografts to Cis-Pt treatment increasing reduction of tumor growth and invasiveness.

## Methods

### In silico analysis of the expression of CA IX and CA XII in HNSCC

For CA IX and CA XII mRNA expression analysis in HNSCC patients the Genomics Analysis and Visualization platform (R2: Genomics analysis and visualization platform; http://r2.amc.nl) was used. The analysis was performed with the following datasets: GSE18674 which includes 22 human normal tissues and GSE42743 which includes 103 oral cavity tumors from Stanford University School of Medicine, Stanford Cancer Center (Standford, CA, USA). The correlation was assessed by one-way analysis of variance (ANOVA), through the R2 platform and presented in box plots.

### Cell lines and culture conditions

The head and neck squamous carcinoma cell lines, FaDu and SCC-011, came from the American Type Culture Collection (ATCC, Manassas, VA) and Cellosaurus JHU-011 (CVCL_5986).

FaDu cells were cultured in Dulbecco Minimum Essential Medium (DMEM) whereas SCC-011 cells in the Roswell Park Memorial Institute (RPMI), supplemented with 10% fetal bovine serum (FBS) and 1% L-glutamine-penicillin–streptomycin and grown at 37 °C with 5% CO_2_.

All experiments were performed by growing FaDu and SCC-011 cells both in normoxic (21% O_2_) and in hypoxic conditions (1% O_2_). Hypoxia was attained in a modular incubator chamber (Stem Cell, Catalog #27,310). The chamber was flooded with the hypoxic gas mixture for 7 min and then sealed and stored in an incubator at 37 °C in 5% CO_2_. The normoxic control was stored in the same incubator for the same amount of time [15].

### Cell viability assay

The viability of FaDu and SCC-011 cells (4 × 10^3^ cells/well, 96-well plates), treated with SLC-0111 (100 µM) alone or in combination with different concentrations of Cis-Pt, was assessed with CellTiter 96 AQueous One Solution Cell Proliferation Assay (Promega BioSciences Inc., Fitchburg, WI, USA) using 3-(4,5-dimethylthiazol-2yl)-5-(3-carboxymethoxy-phenyl)-2-(4-sulfophenyl)-2H tetrazolium (MTS), and according to the manufacturer’s instructions [16].

### SLC-0111 inhibitor

CA IX/XII inhibitor SLC-0111, developed in the laboratory of Professor Claudiu T Supuran (NEUROFARBA Department, University of Florence, Italy) and previously described [17], was used at 100 µM dose alone or in combination with Cis-Pt to evaluate a potential enhanced response of HNSCC cells to conventional chemotherapeutics.

### Cell migration assay

Cell migration was performed as previously reported using 24-well Boyden chambers (Corning, NY) with inserts of polycarbonate membranes (8 µm pores). FaDu and SCC-011 cells (0.5 × 10^5^ /well) were re-suspended in 100 µL of serum-free medium in the presence or absence of Cis-Pt and SLC-0111 (100 µM) and seeded in the upper chamber. After the addition of 10% FBS in the lower chamber as chemo-attractants, the trans-wells were put in a humidified incubator in 5% CO_2_ for 24 h at 37 °C. The non-migrated cells were removed with cotton swabs, whereas the cells that had migrated were visualized by staining the membrane with 0.1% crystal violet in 25% methanol.

10 random fields/filter were counted under a phase contrast microscope (Leica) and images were captured using a digital camera (Canon). All experiments were performed at least three times.

All the results are expressed as the percentage of migrating cells considering the vehicle control sample as 100% [18, 19].

### Cell invasion assay

The invasion assay was performed using the Boyden chamber with membranes (8 µm pores) coated with 50 µL of diluted Matrigel (1:5 in PBS) (Corning, NY, USA). FaDu and SCC-011 cells (1 × 10^5^ /100 µL serum-free medium per well) were harvested, suspended in serum free medium alone or containing Cis-Pt (1 µM) and SLC-0111 (100 µM) and placed in the top chamber. In the lower chamber, a medium containing 10% FBS was added and used as chemo-attractant. Cells were allowed 72 h to invade in a humidified incubator with 5% CO_2_ at 37 °C. To visualize and analyze invading cells, the same experimental procedure described above for cell migration assay was performed. All experiments were performed at least three times [20, 21].

### Spheroid formation assay of HNSCC Cells

FaDu and SCC-011 cells (100 × 10^3^ cell/well) were seeded in ultra-low attachment 6-multiwell-plates (Corning) and grown in spheroid medium containing serum-free DMEM supplemented with B27 (1X), bFGF (20 ng/mL) EGF (10 ng/mL). Cells were incubated at 37 °C with 5% CO_2_ for 7 days. Spheroid formation was analyzed under a phase-contrast microscopy and the size and number of formed spheroids were calculated using ImageJ [22].

### 3D invasion assay

FaDu and SCC-011-spheroids formed, as previously described, were embedded with a matrigel mixture in a ratio 1:1 with spheroid medium. After the spheroids were embedded, cell invasion out of the spheroids was monitored each 24 h. Representative images were acquired and sprouting length was calculated using ImageJ. All images were acquired with an inverted light microscope at 10 × magnification. Data is expressed as mean ± standard deviation of the relative invasive area of the spheroids after 48 h of hypoxia (1% O_2_) [23].

### Clonogenic assay

FaDu and SCC-011 cells (500 cells/well, six-well plates) were cultured with SLC-0111 (100 µM) alone or in combination with Cis-Pt (1 µM) at 37° for 14 days. After washes with DPBS, cells were fixed and stained with 0.1% crystal violet in 25% methanol. Following 30 min at RT, culture dishes were washed with DPBS and colonies were photographed. 1% Sodium Dodecyl Sulphate was added on the cells perfectly washed, in order to induce crystal violet dissolution. Absorbance was recorded at 490 nm by a 96-well-plate ELISA reader [24].

### Cell lysate preparation and western blot analysis

An equal amount of proteins from cells were separated by 4–12% SDS-PAGE and were transferred to a nitrocellulose membrane. Blots were blocked for 1 h with 5% non-fat dry milk and then incubated over night with the following primary antibodies: HIF-1α (BD, Biosciences), CA IX (R&D), CA XII (Santa Cruz), anti-SOX-2 and anti Nanog (CST-9093; Cell Signaling Technology Inc), anti-N-cadherin, anti-E-Cadherin, anti-β-catenin (CST-9782; Cell Signaling Technology Inc), anti-Pro-Caspase-3, anti-cleaved-caspase-3, anti-PARP, anti-cleaved-PARP (CST-9915; Cell Signaling Technology Inc), anti-Stat3 (CST-9139), anti-pStat3 (CST-9138), anti-AKT (CST-9272), anti-pAKT (CST-9271), anti-ERK (CST-9102), anti-pERK (CST-9101), MMP-2 (CST-33437), anti-GADPH (Sigma-G8795), anti-Tubulin (Santa Cruz) and anti-Actin (Sigma-A4700). After washing with 0.1% Tween-20 in PBS, the filters were incubated with their respective secondary antibodies for 1 h and analyzed using the ECL system. Densitometric analyses were performed on at least two different expositions to assure the linearity of each acquisition using ImageJ software (v1.46r) [25, 26].

### Animals

All experimental procedures complied with the European Communities Council directives (2010/63/EU) and national regulations (D.L. 116/92) and were performed in accordance with National Institutes of Health (NIH) recommendations. The present study was approved by the Italian Ministry of Health (authorization number 932/2018-PR). All procedures were performed according to FELASA guidelines for welfare of experimental animals and all imaging acquisition, as well as intravenous injections of fluorescent probes and orthotopic implantations, were performed under general anesthesia with isoflurane (2%) in oxygen (0.8 Lt/min). Each of the imaging workstations used has a heating system to maintain body temperature during the exams, and a system for physiological monitoring (rectal temperature, heart and respiratory rates). All efforts were made to minimize animal suffering and the number of animals necessary to produce reliable results.

FOXN1^NU^ nude mice were subcutaneously injected in the right flank with 2 × 10^6^ FaDu cells, resuspended in 0.1 ml of 1:1 mix of physiological saline and Matrigel. Once tumors became palpable (established), approximately 50 mm^3^ [volume = 0.5 × long diameter × (short diameter)^2^] [27], nude mice were randomized into 4 treatment groups (3 mice for each group): *i*) Cis-Pt – treated with i.p. injection of 3 mg/kg of Cis-Pt, in a total volume of 50 µL, every other day for 3 weeks; *ii*) SLC-0111 treated with *per os* administration, via oral gavage, of SLC at 100 mg/kg in a total volume of 100 µL, once per day, five days per week for 3 weeks, the oral formulation of SLC-0111 consisted of 55.6% PEG400/11.1% ethanol/33.3% water [28]; *iii*) combined therapy with Cis-Pt and SLC-0111 at the aforementioned dosages and administration schemes; *iv*) vehicle group receiving 100 µL of phosphate buffered saline i.p. every other day for 3 weeks. Mice were checked and weighted twice per week, and tumor mass were measured with a manual caliper during these operations. In order to develop an HNSCC orthotopic model 2 × 10^6^ FaDu cells, resuspended in 0.1 ml of 1:1 mix of physiological saline and Matrigel, was gently injected in the left lingual muscular belly with the animal under general anesthesia. For the intravenous injection of fluorescent probes Annexin Vivo 750 and ProSense 750, animals were intravenously catheterized in one lateral tail vein of mice maintained under isofluorane anesthesia, using a catheter mounting a 30G needle. Then 2 nmoles of the probe were slowly injected and the catheter was further flushed with 10–20 µL of sterile saline. At the end of imaging studies mice were euthanized. Treatment schedule is schematized in Fig. 7A.

### Fluorescent imaging

All mice were maintained on a diet with a purified, alfalfa-free rodent chow for 15 days before fluorescence imaging to minimize fluorescence in the gut. Near-infrared fluorescent molecular imaging was performed with Annexin Vivo 750 and ProSense 750 using FMT4000 (Perkin-Elmer Inc., Waltham, MA, USA). 2 nmol of Annexin Vivo 750 were administrated to each HNSCC s.c. xenografts and FRI was used to study *in vivo* xenografts accumulation of after 2 and 24 h, and *ex vivo* in the surgically harvested primary tumor mass. 2 nmol of Prosense 750 were administrated to each HNSCC orthotopic xenografts and FMT of head, neck and thorax was used to study accumulation in the orthotopic tumor, neck lymph nodes and lungs. The tongue including the orthotopic tumor and the before mentioned organs where surgically harvested and studied with FRI. Mice were placed in dorsal recumbency to study the orthotopic tumor, neck lymph nodes and lungs, *in vivo*, and in lateral recumbency for xenograft studies. At the end of the imaging acquisition mice were euthanized while under anesthesia and the aforementioned organs were harvested for *ex vivo* evaluation. Images were reconstructed with FMT system software (TrueQuantTM v4.0) from PerkinElmer (Waltham, MA). For FRI an elliptical region of interest (ROI) was designed over the xenograft excluding as much as possible of surrounding normal tissues, the total counts/energy were recorded for each animal at 2 and 24 h. Similar ROI was designed over explanted tissues for *ex vivo* analysis.

### Statistical analysis

Data were analyzed with GraphPad Prism statistical software 8.0 (GraphPad Software, La Jolla, CA, USA). *In vitro* results were obtained from at least three independent experiments and are expressed as means ± standard deviation and significance was determined using Student’s t test.

A *p* value < 0.05 was considered statistically significant. For *in vivo* results, normality was tested with a Shapiro–Wilk’s test, and parametric or non-parametric tests were chosen accordingly. Tumor volumes and bodyweights were studied over time by applying a mixed model analysis of variance for repeated measures (RM-ANOVA) with Geisser-Greenhouse correction, hence studying the effect of time, of the treatment group and of their interaction. Post hoc, Fisher’s LSD test for multiple comparisons was applied between groups. The FRI counts/energy, *in vivo* at 2 and 24 h and *ex vivo*, between treatment groups were tested with a Kruskal–Wallis test, and post hoc with a Dunn’s test for multiple comparisons.

## Results

### Hypoxic TME increases Cis-Pt resistance and CA IX/XII expression levels in HNSCC

**Fig. 1:**
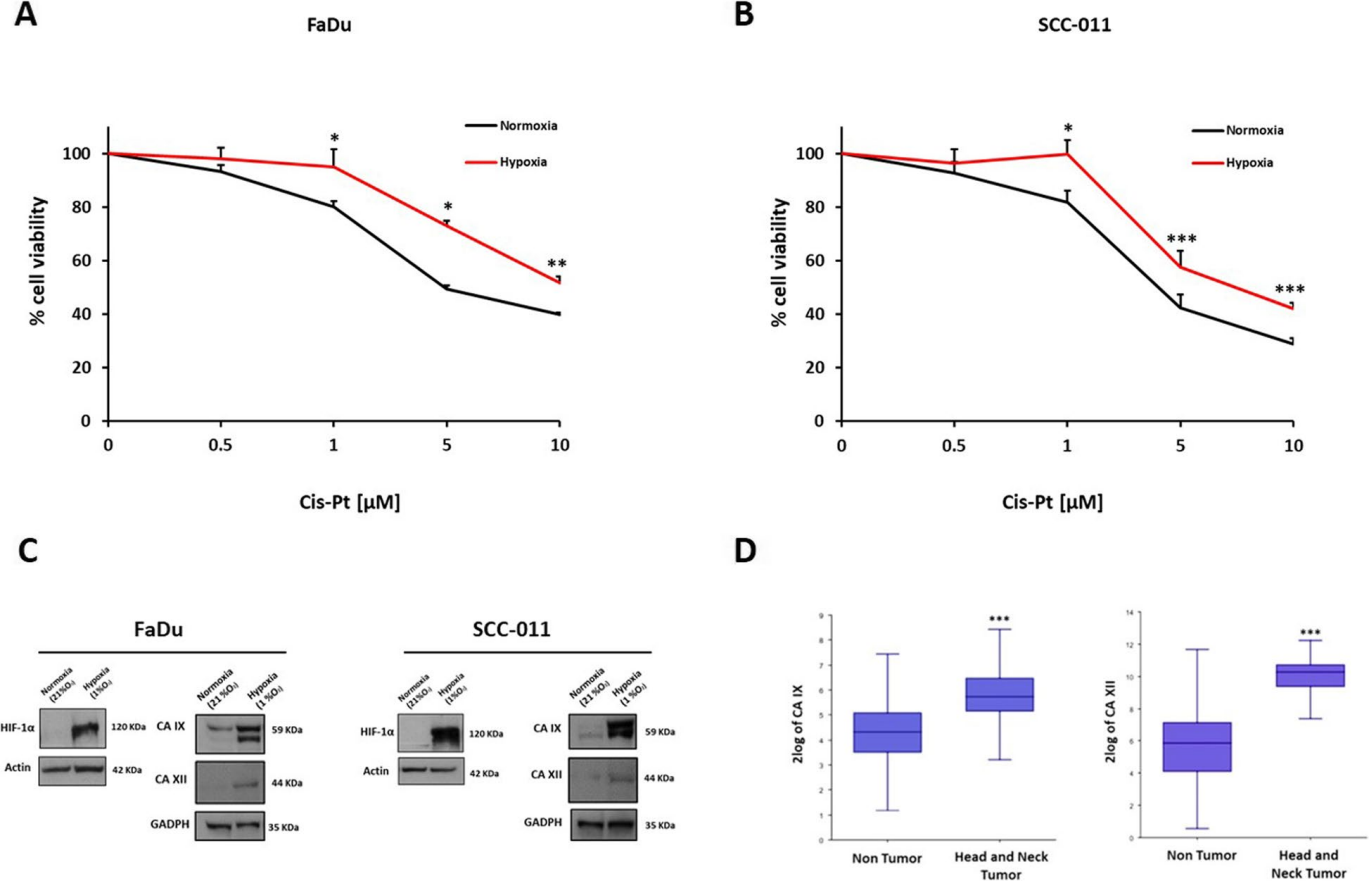
Hypoxic TME increases Cis-Pt resistance and CA IX/XII expression levels in HNSCC. **A-B** Cell viability analysis by MTT assay of FaDu and SCC-011 cells grown in normoxic (21% O_2_) and hypoxic (1% O_2_) conditions and incubated with increasing concentrations of Cis-Pt (0–10 µM) for 72 h. All the data are expressed as percentage of viable cells, considering the untreated control cells as 100%. Bars depict mean ± SD of three independent experiments. (***p < 0.001; **p < 0.01; *p < 0.1). **C** HIF-1α, CA IX and CA XII protein levels were analyzed by western blot analysis in FaDu and SCC-011 cell lines grown under normoxia (21% O_2_) and hypoxia (1% O_2_) for 72 h. Actin and GADPH were used as loading control. Representative data from one of three experiments are shown. **D** For CA IX and CA XII mRNA expression analysis in HNSCC patients the Genomics Analysis and Visualization platform (R2: Genomics analysis and visualization platform; http://r2.amc.nl) was used. The analysis was performed with the following datasets: GSE18674 which includes 22 human normal tissues and GSE42743 which includes 103 oral cavity tumors. The correlation was assessed by one-way analysis of variance (ANOVA), through the R2 platform and presented in box plots. (***p < 0.0001)

The first-line chemotherapy agent for treatment of HNSCC is Cis-Pt and the hypoxic microenvironment is the prime factor underlying tumor insensitivity to this drug. In order to evaluate how hypoxic microenvironment impacts on sensibility of HNSCC cells to Cis-Pt, FaDu and SCC-011 cell lines were grown in normoxic (21% O_2_) and hypoxic (1% O_2_) conditions and incubated with increasing concentrations of Cis-Pt (0–10 µM) for 72 h, then cell viability was assessed by MTT assay. As shown in Fig. 1A and B, hypoxia caused a reduction of Cis-Pt cytotoxicity in both HNSCC cell lines, indicating that low levels of O_2_ induced resistance to treatments *in vitro*.

To demonstrate the induction of hypoxia the expression levels of hypoxia-inducible factor 1-alpha (HIF-1α) were analyzed in both HNSCC lines grown under normoxic (21% O_2_) and hypoxic (1% O_2_) conditions. As expected the highest levels of HIF-1α were found in HNSCC cells grown in hypoxia (Fig. 1C). According to the hypoxia-induced expression of CA IX/XII enzyme reported in solid tumors, we observed an upregulation of CA IX/XII in FaDu and SCC-011 cells grown in hypoxia in comparison with cells grown in normoxia (Fig. 1C). Furthermore, we evaluated the expression of these enzymes in two public datasets: one including 22 normal tissues and another 103 HNSCC samples. The analysis showed that the tumors expressed higher levels of mRNA CA IX/XII respect to normal tissue (*p* < 0.0001) (Fig. 1D).

### Inhibition of CA IX/XII by SLC-0111 sensitizes HNSCC cells to Cis-Pt

**Fig. 2:**
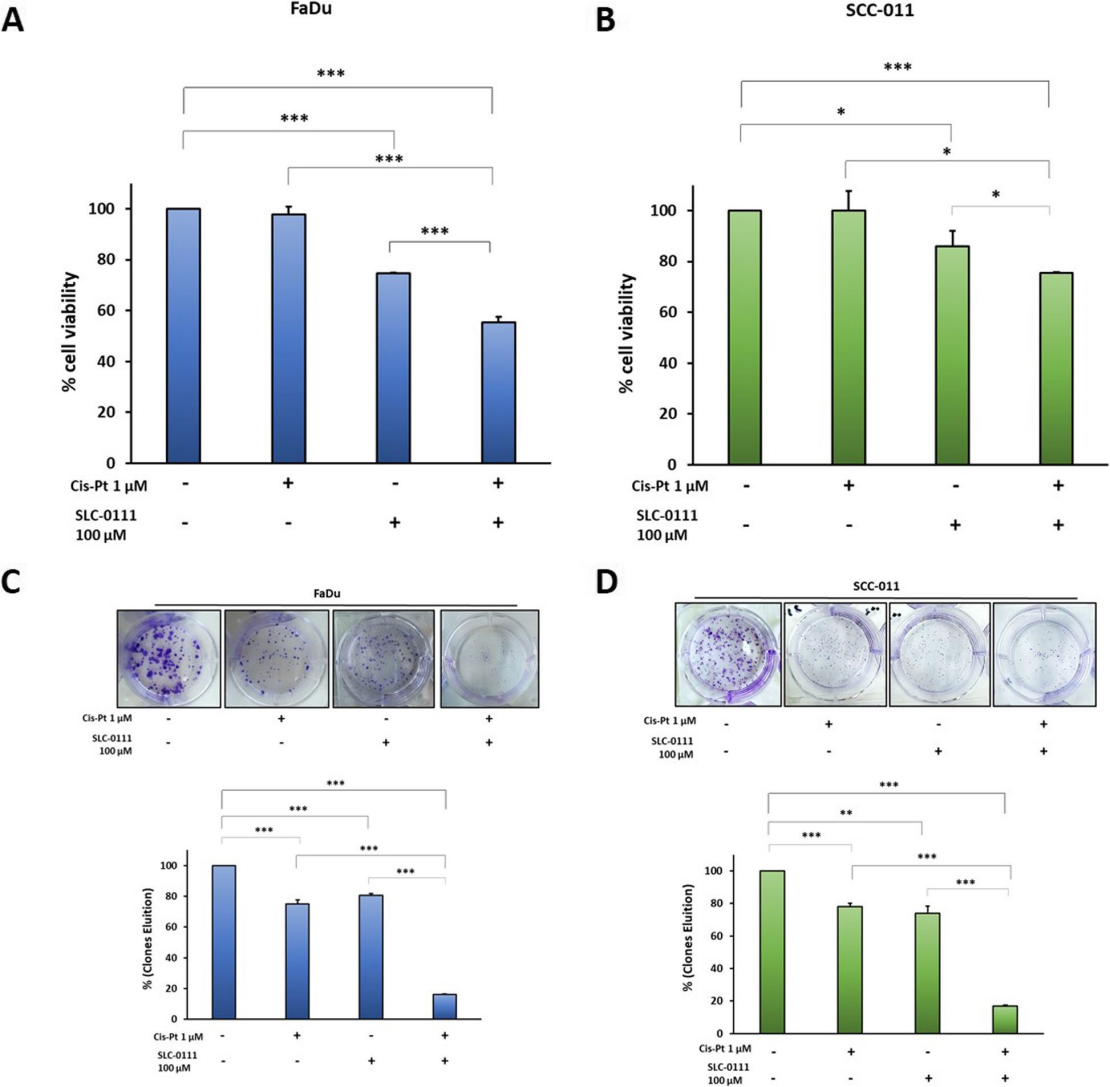
Inhibition of CA IX/XII by SLC-0111 sensitizes HNSCC cells to Cis-Pt. Cell viability analysis of FaDu (**A**) and SCC-011 (**B**) cells grown under hypoxic conditions (1% O_2_) and treated with Cis-Pt (1 µM), SLC-0111 (100 µM) and combination of the two drugs for 72 h. Cell survival of FaDu (**C**) and SCC-011 (**D**) cells grown under hypoxic conditions (1% O_2_) and treated with Cis-Pt (1 µM), SLC-0111 (100 µM) and combination of the two drugs was assessed by colony formation assay cells. All the data are expressed as percentage considering the untreated control cells as 100%. Bars depict mean ± SD of three independent experiments. (***p < 0.0001; ** *p* < 0.001; *p < 0.01)

To investigate whether the inhibition of hypoxia-induced enzymes CA IX/XII could augment the efficacy of low doses of Cis-Pt against HNSCC, FaDu and SCC-011 cell lines were grown under hypoxic conditions and treated with Cis-Pt (1 µM), the specific CAIX/XII inhibitor SLC-0111 (100 µM) and combination of the two drugs for 72 h. Analysis of cell viability showed that 1 µM of Cis-Pt did not have any significant effect in both cell lines, whereas 100 µM of SLC-0111 caused a significant viability reduction of 26% (*p* = 0.0001) and 15% (*p* = 0.01) in FaDu and SCC-011 cell lines, respectively (Fig. 2A and B). The addition of SLC-0111 to Cis-Pt treatment had a higher inhibitory effect in comparison to each drug alone (FaDu *p* < 0.0001; SCC-011 *p* < 0.0001). Furthermore, cells, treated as above, were left to grow for 10 days and a clonogenic assay was performed. As shown (Fig. 2C and D), both Cis-Pt and SLC-0111, used as single agents, caused a significant reduction of clone proliferation in FaDu and SCC-011 cell lines. Notably, the number of clones was drastically reduced in the presence of the combinatorial therapy.

### The combination of SLC-0111 and Cis-Pt drastically reduces HNSCC cell migration and invasion

**Fig. 3:**
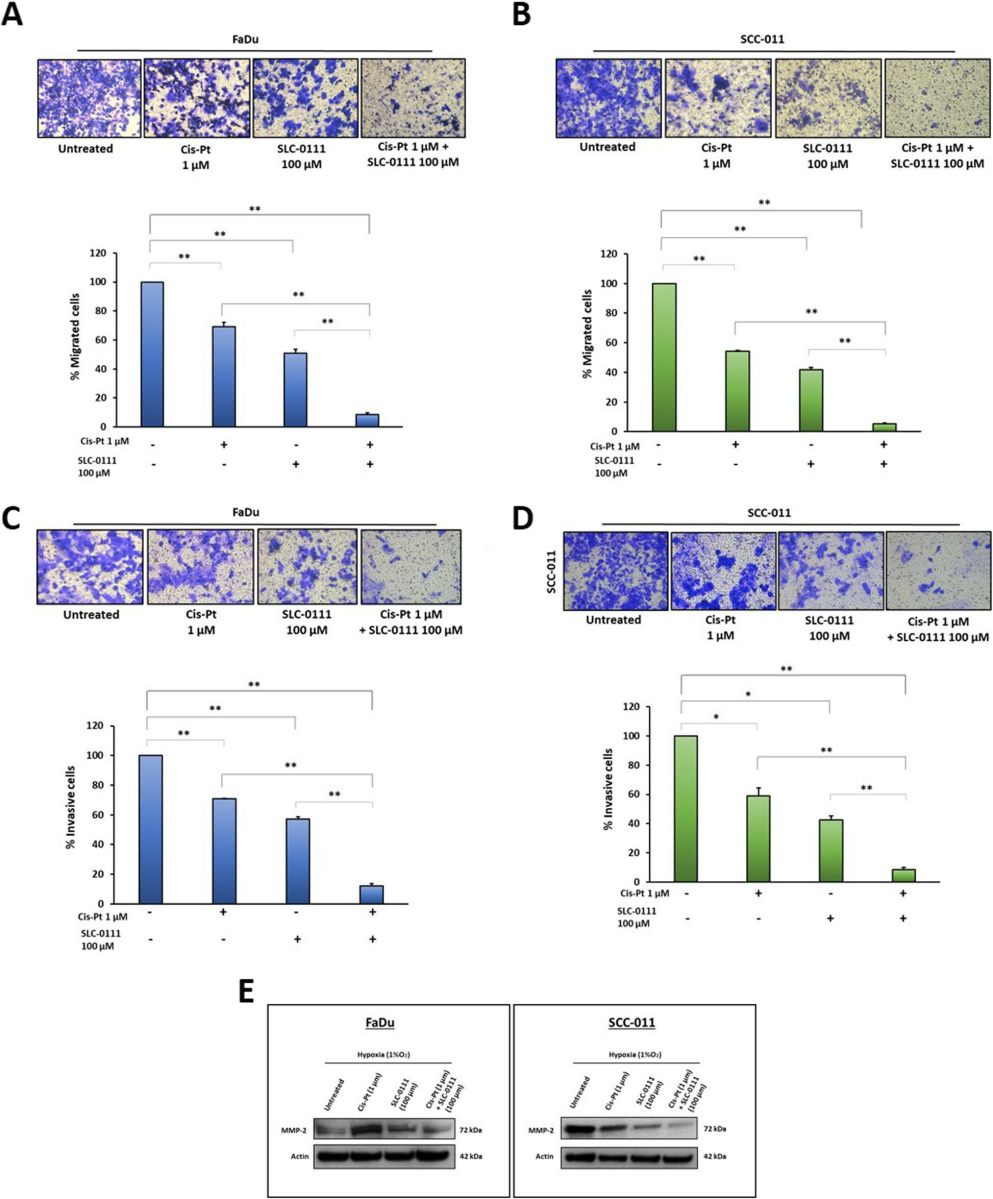
The combination of SLC-0111 and Cis-Pt drastically reduces HNSCC cell migration and invasion. **A**-**B** Migration assay of FaDu and SCC-011 cells grown under hypoxia (1%O_2_) and treated with Cis-Pt (1 µM), SLC-0111 (100 µM) and combination of the two drugs for 24 h was performed using trans-well chamber containing inserts of polycarbonate membranes with 8 μm pores. In the lower chamber, a medium containing 10% FBS was added and used as chemo-attractant. **C-D** Invasion assay of FaDu and SCC-011 cells was performed for 72 h as described above on membranes coated with Matrigel. Representative photographs of at least three different experiments are shown. The results are expressed as percent of migrating and invading cells considering the untreated control as 100%. Bars depict mean ± SD of three independent experiments (** *p* < 0.0001; * *p* < 0.001). **E** Western blot analysis of expression levels of MMP-2 in FaDu and SCC-011 grown under hypoxia (1% O_2_) and treated with Cis-Pt (1 µM), SLC-0111 (100 µM) and combination of the two drugs for 72 h. Actin was used as loading control

The consequences of failure of chemotherapy treatment are relapse of disease and tumor cells metastatic spread, therefore we investigated whether the addition of SLC-0111 to Cis-Pt could improve the effect of Cis-Pt on HNSCC cell migration and invasion. To demonstrate this, we performed migration and invasion assays using trans-well chambers that were coated with Matrigel to assess cell invasiveness. 10% FBS was added to the lower chamber as chemoattractant and experiments were carried out under hypoxic conditions. We observed that both Cis-Pt and SLC-0111 reduced cell migration at 24 h when used as single agents in both HNSCC cell lines, with a greater effect in SCC-011 cell line, in comparison with untreated cells (FaDu: reduction of 31% and 50% after Cis-Pt and SLC-011 treatment, respectively; SCC-011: reduction of 46% and 59% after Cis-Pt and SLC-011 treatment, respectively; (*p* < 0.0001) (Fig. 3A and B). The addition of SLC-0111 to Cis-Pt greatly amplified its inhibitory effect on tumor cell migration (FaDu: reduction of 92%, SCC-011: reduction of 95%; p < 0.0001) (Fig. 3A and B). Similar results were obtained when HNSCC cells were allowed to invade Matrigel for 72 h. Indeed, single treatment with Cis-Pt and SLC-0111 decreased the invasiveness of HNSCC cells (FaDu: reduction of 30% and 43% after Cis-Pt and SLC-011 treatment, respectively; SCC-011**:** reduction of 42% and 58% after Cis-Pt and SLC-0111 treatment, respectively; *p* = 0.001), whereas the combination of the two drugs drastically blocked it (FaDu: reduction of 88%, SCC-011: reduction of 92%; *p* ≤ 0.0001) (Fig. 3C and D). In addition, as shown in Fig. 3E, the expression levels of the matrix-degrading enzyme Metalloproteinase-2 (MMP-2), involved in promoting tumor cell invasion, were more reduced after treatment with SLC-0111 and cisplatin than after single drug treatments.

### The combination of SLC-0111 and Cis-Pt inhibits tumorsphere formation and invasion

**Fig. 4:**
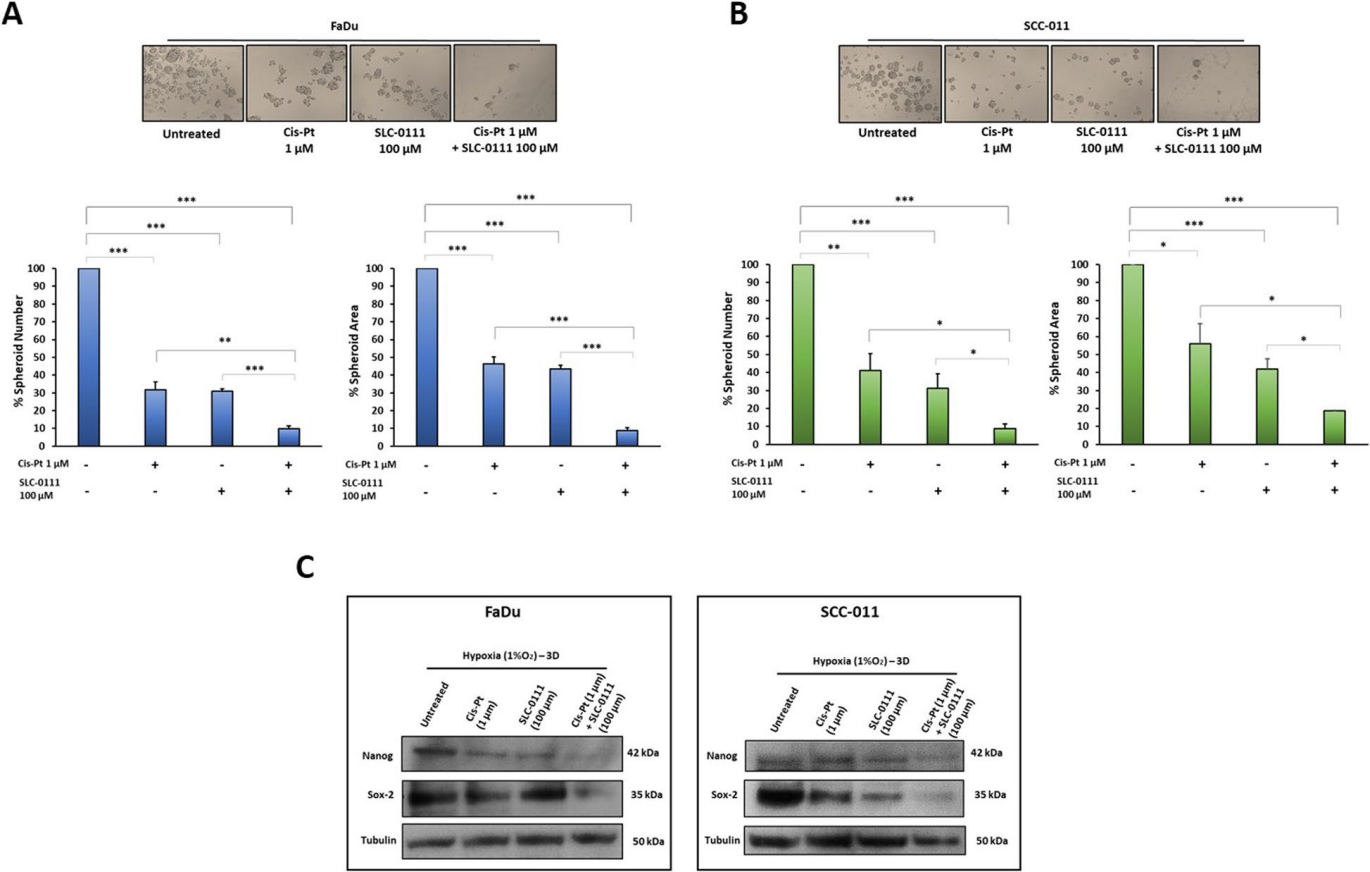
Effect of combinatorial treatment with SLC-0111 and Cis-Pt on tumorsphere formation and stemness. FaDu (**A**) and SCC-011 (**B**) cells were seeded in Ultra-Low attachment multiwell-plates and grown in serum free DMEM supplemented with B27, bFGF (20 ng/mL) and EGF (10 ng/mL) in hypoxic conditions for 7 days. HNSCC cells were treated with Cis-Pt (1 µM), SLC-0111 (100 µM) and combination of the two drugs for 72 h. Spheroid formation was analyzed under a phase-contrast microscopy and size and number of formed spheroids was calculated using ImageJ. All the data are expressed as percentage considering the untreated control cells as 100%. Bars depict mean ± SD of three independent experiments (*** *p* < 0.0001; ** *p* < 0.001; *p < 0.01). **C** Western blot analysis of expression levels of stemness markers Nanog and Sox-2 in FaDu and SCC-011 grown under hypoxia (1% O_2_) and treated with Cis-Pt (1 µM), SLC-0111 (100 µM) and combination of the two drugs for 72 h. Tubulin was used as loading control. Representative data from one of three experiments are shown

**Fig. 5:**
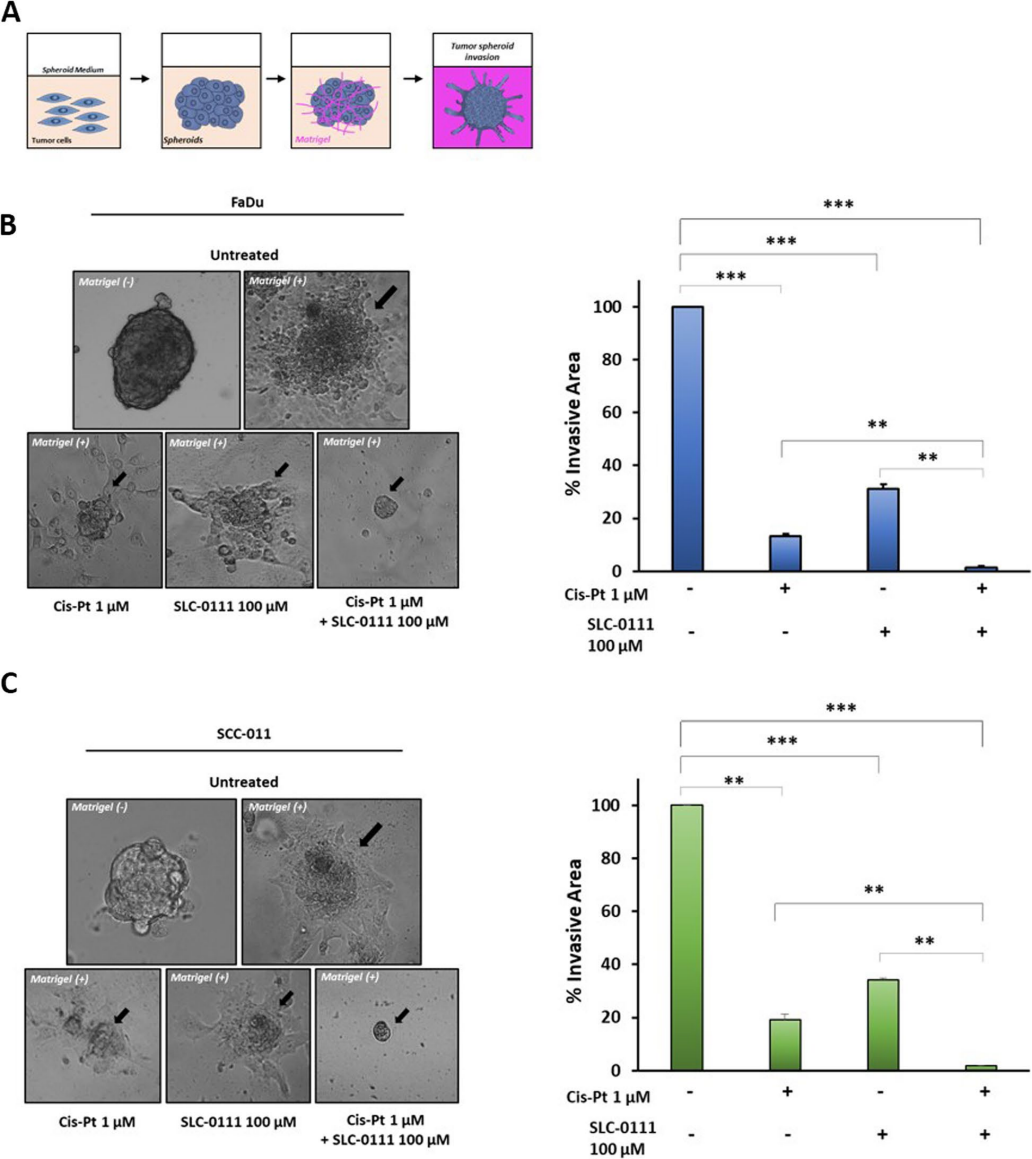
Effect of combinatorial treatment with SLC-0111 and Cis-Pt on tumorsphere invasion. FaDu (**A**) and SCC-011 (**B**) spheroids were embedded in matrigel and grown under hypoxia (1% O_2_) and treated with Cis-Pt (1 µM), SLC-0111 (100 µM) and combination of the two drugs for 72 h. Images were acquired and invasive area was calculated using ImageJ. Representative photographs of at least three different experiments are shown. The results are expressed as percent of invasive area considering the untreated control as 100%. Bars depict mean ± SD of three independent experiment. (***p < 0.0001; ** *p* < 0.001)

Cancer stem cells (CSCs) are a sub-population of cells within cancer tissues with tumor initiation, drug resistance and metastasis properties. Hypoxic/acidic microenvironment is considered a driver of the ability of cancer cells to acquire stemness characteristics and CA IX overexpression is reported to be correlated with this [9]. Stemness status of HNSCC cells was evaluated by their ability to form tumorspheres *in vitro*, culturing cancer cells onto ultra-low attachment surface in serum-free media under the supplementation with growth factors [29]. Treatment of FaDu and SCC-011 cells with Cis-Pt or SLC-0111 reduced their ability to form non-adherent spheroids with stemness characteristics in hypoxic TME, decreasing both their number and area (FaDu: Cis-Pt 69% and 54%; SLC-011 69% and 57%, respectively; p < 0.0001) (SCC-011: Cis-Pt 59% and 44%, respectively, *p* = 0.01 and *p* = 0.001; SLC-0111 69% and 59%, respectively, *p* ≤ 0.0001) compared to untreated cells (Fig. 4A and B). When cells were treated with combination of Cis-Pt and SLC-0111 the growth of spheroids was strongly reduced as amount (FaDu reduction of 90%; SCC-011 91.2%; p < 0.0001) and size (FaDu reduction of 91%; SCC-011 82%; *p* < 0.0001) in comparison to each single treatment (Fig. 4A and B). In addition, as shown in Fig. 4C, the expression levels of Nanog and Sox-2 stemness markers were drastically reduced after combinatory treatment. Next, to evaluate the capability of the tumor cells organized into a 3D structure, mimicking a tumor micro-region with stemness features, to invade matrix they were embedded in Matrigel for 72 h (Fig. 5A). Both HNSCC cell lines showed numerous and long protrusions invading Matrigel in untreated conditions whereas the addition of Cis-Pt and SLC-0111 caused a significant reduction of the spikes. Notably, the combination of two drugs completely abolished the invasiveness capability of the spheroids (Fig. 5B and C).

Taken together these results demonstrated that addition of the CA IX/XII inhibitor SLC-0111 to Cis-Pt treatment is able to hamper not only the migration and invasion of cancer cells but also of cancer stem cells characterized by a greater resistance to chemotherapy treatments.

### SLC-0111 in combination with Cis-Pt prevents activation of STAT3, AKT, ERK and EMT program

**Fig. 6:**
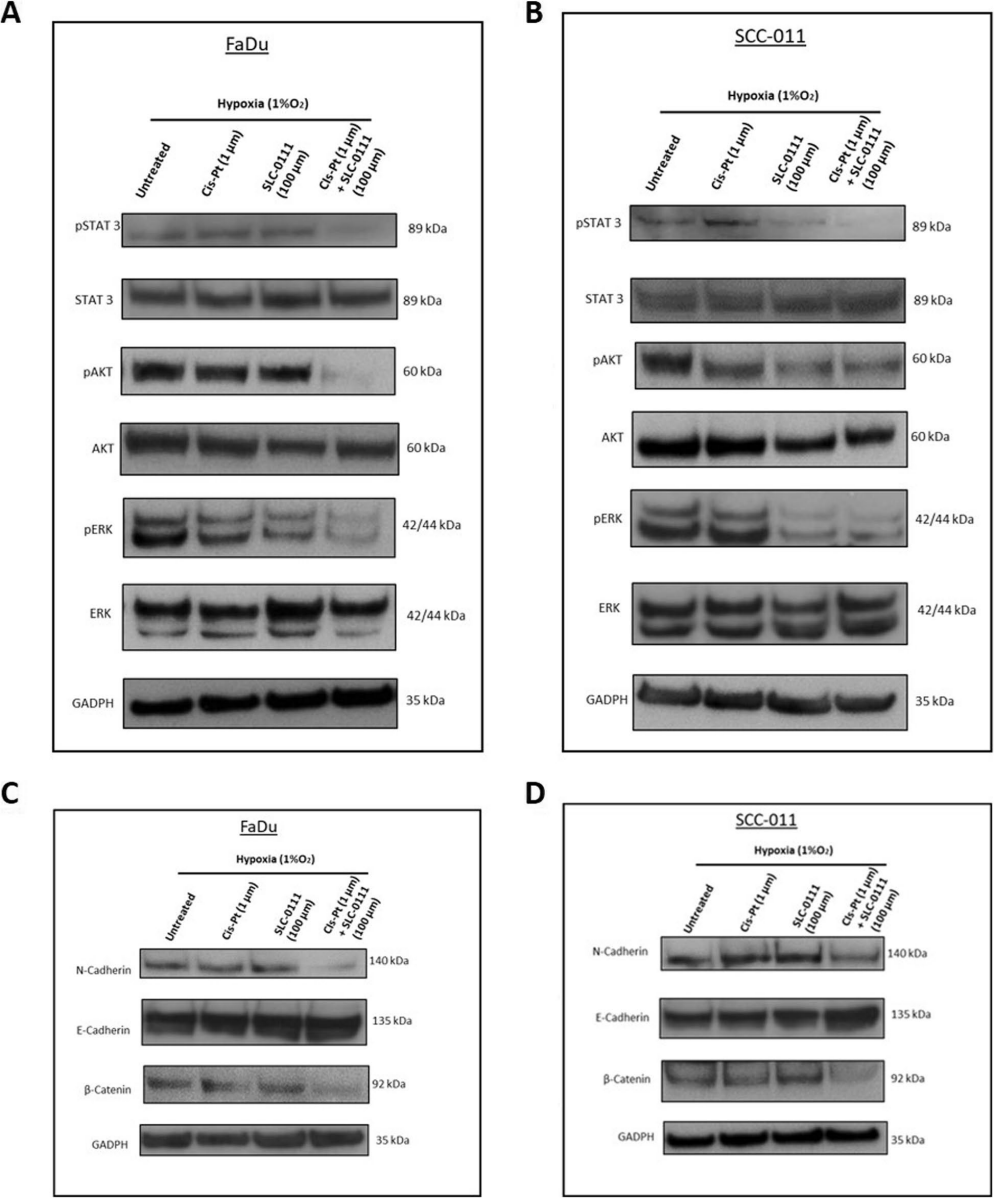
Effect of combinatorial treatment with SLC-0111 and Cis-Pt on STAT3, AKT, ERK and EMT program activation. **A-B** Analysis by western blot of expression levels of pSTAT3/STAT3, pAKT/AKT and pERK/ERK. **C-D** Analysis by western blot of expression levels of epithelial (E-Cadherin) and mesenchymal markers (N-Cadherin and β-Catenin). FaDu and SCC-011 cells were grown under hypoxia (1%O_2_) and treated with Cis-Pt (1 µM), SLC-0111 (100 µM) and combination of the two drugs for 72 h. Equal loading was confirmed by immunoblot with anti-GADPH antibody. Representative images are shown

Next, we analyzed the main signaling pathways that play a crucial role in the hallmarks of cancer such as the activation of STAT3 (Signal Transducer and Activator of Transcription 3), AKT (Protein Kinase B) and ERK (Extracellular signal-regulated Kinase). When both cell lines were treated with Cis-Pt plus SLC-0111, a stronger reduction of phosphorylated form of STAT3, AKT and ERK was observed in comparison with that observed in cells untreated or treated with Cis-Pt or SLC-0111 alone (Fig. 6A and B). In SCC-011 cells also SLC-0111 as single treatment showed an inhibitory effect on expression levels of phosphorylated proteins (Fig. 6B). Furthermore, we investigated whether the inhibition of CA IX/CAXII could prevent the Epithelial-Mesenchymal Transition (EMT) program in HNSCC cell lines. We observed that the expression levels of two mesenchymal markers such as N-cadherin and β-catenin were reduced, when tumor cells were treated with SLC-0111 and Cis-Pt combinatorial approach, conversely the epithelial marker E-cadherin was upregulated thus demonstrating a reversal of EMT (Fig. 6C and D).

### SLC-0111 increases the effect of Cis-Pt in vivo

**Fig. 7:**
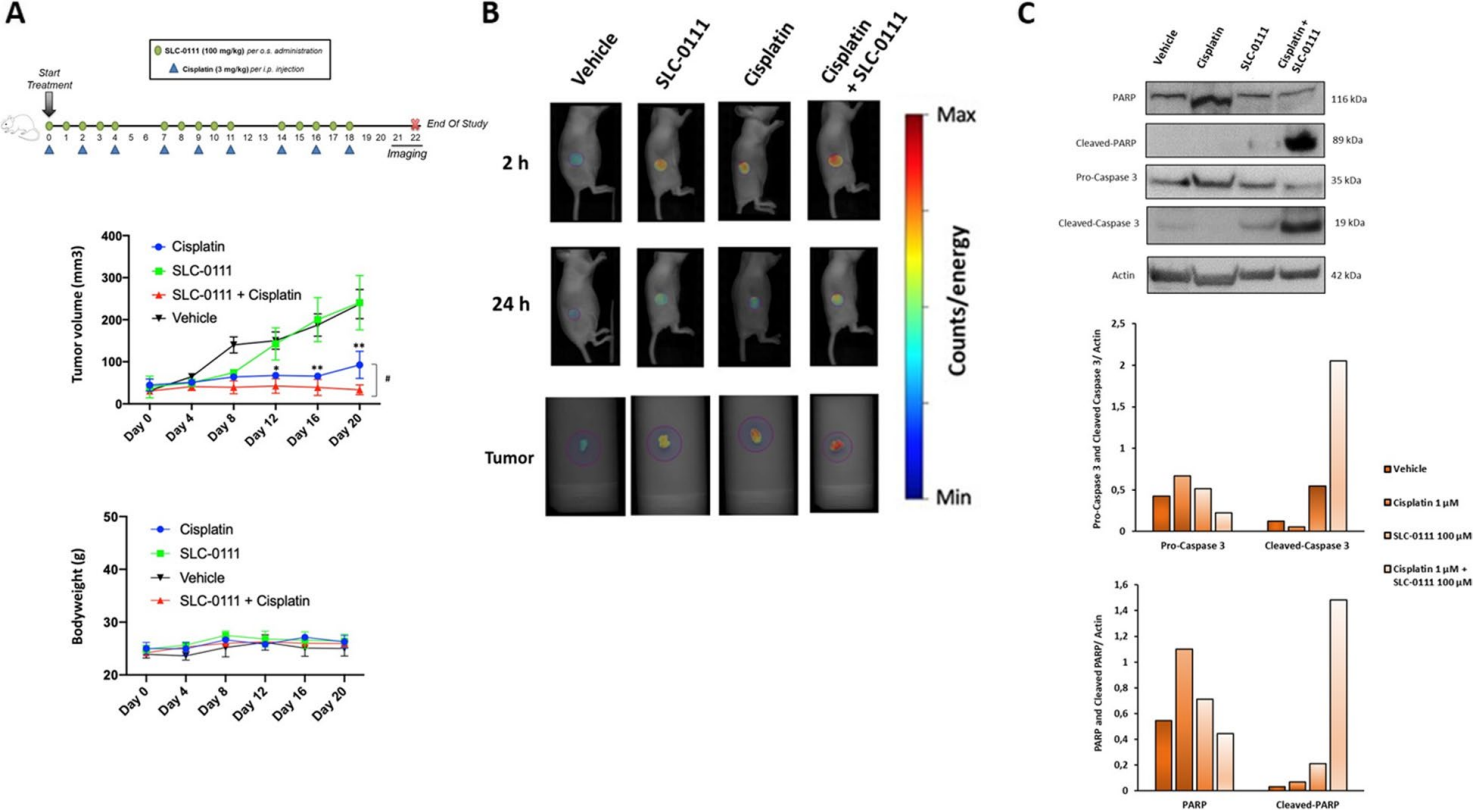
SLC-0111 increases the effect of Cis-Pt on tumor growth *in vivo. ***A** Nude mice bearing FaDu xenografts, after tumor establishment (50 mm^3^), were treated with vehicle, SLC-0111 (100 mg/kg per os administration, via oral gavage) or Cis-Pt alone (3 mg/kg per i.p. injection), or the two drugs in combination, as shown in treatment schedule. Tumor growth was monitored by calipers overtime and tumor volume mean ± SD of each group is reported. Mice body-weight was measured at the indicated days and the weight mean ± SD is shown. (*p < 0.0001 relative to vehicle; #*p* < 0.05 combinatorial treatment vs Cis-Pt alone) (**B**) At end of treatments nude mice bearing FaDu xenografts were injected with 2 nmol of Annexin Vivo 750 and subjected to imaging studies using FRI at 2 and 24 h. At end of imaging study, tumors were harvested for ex vivo FRI analysis. Representative images as shown. **C** Lysates from recovered tumors were immunoblotted with antibodies anti pro-caspase-3/cleaved-caspase-3 and PARP/cleaved-PARP. Equal loading was confirmed by immunoblot with anti-Actin antibody. The graphs display the relative quantities of protein expression levels

Given our *in vitro* observations that demonstrated the ability of SLC-0111 to increase the efficacy of Cis-Pt in HNSCC cells, we investigated whether there could be a benefit of this combinatorial therapy also *in vivo* in HNSCC murine models. To this aim, we subcutaneously implanted FaDu cells in athymic nude immunocompromised mice. After tumor establishment (50 mm^3^), mice were treated with vehicle, SLC-0111 (100 mg/kg per os administration, via oral gavage) or Cis-Pt alone (3 mg/kg per i.p. injection), or the two drugs in combination, as shown in treatment schedule schematized in Fig. 7A. The analysis of xenograft tumor volumes showed a significant effect of the treatment group (*P* = 0.001), and also a significant effect of time and of their interaction (*P* = 0.0003 and *P* < 0.0001, respectively; Fig. 7A). SLC-0111 treatment alone was ineffective to reduce tumor growth *in vivo* in comparison to vehicle control. Conversely, xenograft treatment with Cis-Pt, either if administrated alone or in combination with SLC-0111, caused a significant decrease of tumor growth compared to vehicle control, starting from day 7 up to the end of the study. Notably, at this time point (day 20), the addition of SLC-0111 significantly improved the efficacy of Cis-Pt monotherapy (*P* < 0.05) (Fig. 7A).

Regarding body weight, no effect of the treatment group neither of the interaction between group and time was detected (Fig. 7A).

In order to confirm the ability of SLC-0111 to enhance the effect of Cis-Pt *in vivo,* FaDu tumor bearing nude mice were injected with Annexin Vivo 750, a NIR imaging probe to enable *in vivo* visualization of apoptosis, and subjected to imaging studies using FRI at 2 and 24 h. Then, the tumors of the mice were harvested for *ex vivo* FRI analysis. *In vivo* and *ex vivo* images of tumors showed a significantly higher accumulation of Annexin Vivo 750 in tumor of treated mice in comparison with control group (*P* = 0.0048) (Fig. 7B). Notably, the drug combinatorial group showed the highest intensity of Annexin Vivo 750 signal at 2 h (*P* = 0.003) (Fig. 7B). These results were corroborated by *in vitro* findings obtained on untreated and treated FaDu and SCC-011 cell lines, stained with Annexin V/PI and subjected to flow cytometry analysis, that showed a significant increase of apoptosis when they were treated with Cis-Pt plus SLC-0111 respect to the single drugs (Supplementary Fig. 1A and C). Furthermore, immunoblot analysis performed on lysates obtained from FaDu xenografts (Fig. 7C) and FaDu cells as well as SCC-011 cells (Supplementary Fig. 1B and D) showed a reduction of full-length caspase-3 and PARP levels and a strong increase of both cleaved forms after drug combinatory treatment respect to single treatments confirming an increased activation of the apoptotic program. In addition, in agreement with *in vitro* results, a greater reduction in MMP-2 expression levels was observed in xenograft lysates after combinatorial treatment compared with single-drug treatments (Supplementary Fig. 2).

Next, we investigated whether the combined treatment Cis-Pt with SLC-0111 could have any effect on metastatic spread *in vivo*, a preliminary study was carried out to refine the intervention and evaluate its acceptability and feasibility on mice bearing the orthotopic HNSCC xenografts. Mice were subjected after the different drug treatments to *in vivo* imaging using the FRI with ProSense 750, a cathepsin-activatable fluorescent imaging agent, commonly used to detect the metastatic process [30].

**Fig. 8:**
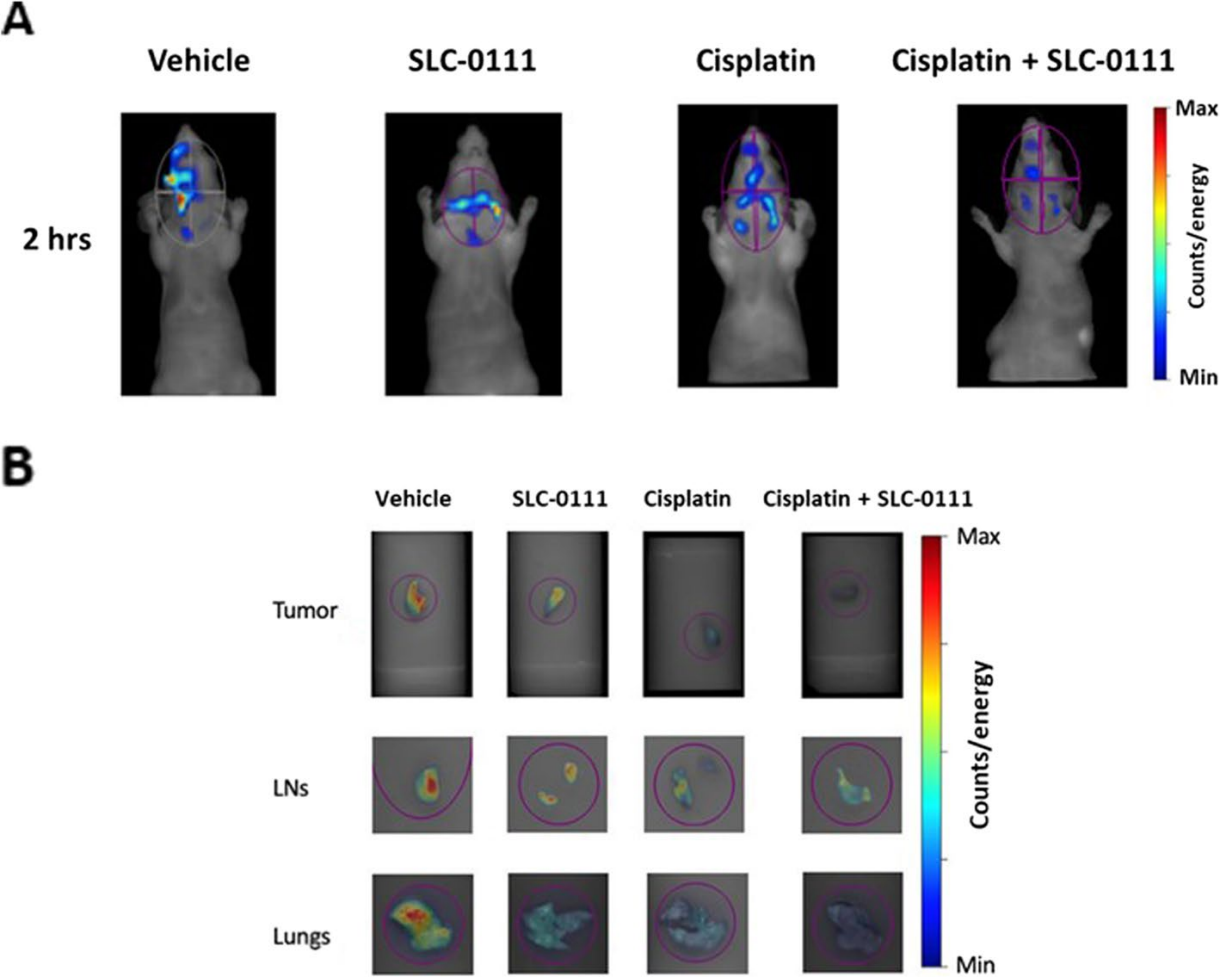
SLC-0111 increases the effect of Cis-Pt on metastatic spread in vivo. **A** Nude mice bearing orthotopic FaDu xenografts were treated with vehicle, SLC-0111 (100 mg/kg per os administration, via oral gavage) or Cis-Pt alone (3 mg/kg per i.p. injection), or the two drugs in combination, as reported in treatment schedule shown in Fig. 7A. At end of treatments FaDu xenografts were injected with 2 nmol of ProSense 750 and subjected to imaging studies using FRI at 2 h. **B** At end of imaging study, tumors, head-neck lymph nodes and lungs were harvested for ex vivo FRI analysis. Representative images as shown

*In vivo* imaging showed an intense NIR fluorescence signal in tumor region that was drastically reduced in the mouse that received combined treatment with SLC-0111 and Cis-Pt compared to animals treated with vehicle or single drugs (Fig. 8A). *Ex vivo* images of tumors, head-neck lymph nodes and lungs showed a greater decrease of ProSense 750 signal following combined treatment (Fig. 8B). These preliminary *in vivo* evidences, together with the results obtained *in vitro*, strongly suggest the potential of SLC-0111 to enhance Cis-Pt effect not only on tumor growth but also on metastatic spread.

## Discussion

Many findings demonstrated that the tumor hypoxic microenvironment contributes to the development of resistance to anticancer therapies including radiotherapy and chemotherapy, immunotherapy and targeted therapy [7]. Furthermore, low levels of O_2_ in TME favor cancer cell migration, invasion, stemness and establishment of secondary metastases [6]. A recent and noteworthy paper by Weiss et al. reported emerging evidences showing how the strict relationship between signaling networks underlying therapy resistance and metastatic progression cooperate to a fatal outcome of cancer [31].

Hypoxia is a common characteristic and a negative prognostic factor in HNSCC and it is correlated with aggressive and invasive phenotype as well as with failure to conventional therapies [32, 33]. Cis-Pt is the standard chemotherapy regimen to treat advanced/metastatic HNSCC although after treatment majority of patients develop drug resistance and disease relapse [2]. It has been reported that the hypoxic TME is the crucial driver in the onset of insensitivity to Cis-Pt [8].

The adaptive changes in cancer cells to lack the oxygen supply are responsible for Cis-Pt resistance. There are two carbonic anhydrase isoforms IX and XII that are highly expressed in hypoxic tumors and both are involved in therapy resistance and metastatic dissemination [9].

Previous studies demonstrated the overexpression of CA IX in HNSCC [34, 35] and its correlation with reduced overall survival and disease-free survival [36]. In this study, we evaluated the efficacy of CA IX and CA XII selective inhibitor SLC-0111, an ureido-substituted benzenesulfonamide small molecule, to sensitize HNSCC cells and animal models to Cis-Pt, thus reducing tumor cell growth and dissemination. This compound entered in phase Ib/II clinical trials in patients with previously treated advanced solid tumors to determine its safety and tolerability and establish the recommended clinical dose [37]. This study, showing that SLC-0111 was better tolerated at the 1000-mg dose compared with the 2000-mg dose, and the PK parameters were similar at these doses, supported 1000 mg/d as the recommended phase II dose (RP2D) [37]. Previous investigations demonstrated the ability of SLC-0111 to increase the effects of chemotherapeutic treatments in cancer cells. In particular, Andreucci et al. showed that this molecule sensitized melanoma cells to decarbazine and temozolomide (TMZ), breast cancer cells to doxorubicin and colorectal cancer cells to 5-fluoracil [11].

A crucial role in cancer resistance to therapies and metastatic dissemination is played by both CSCs and EMT program which exhibit interconnected signaling pathways [38]. High levels of CA IX were found to correlate with both stemness and EMT underlying the aggressiveness of solid tumors [9]. Recently, we showed that inhibition of CA IX/XII caused a significant reduction of CSCs and hampered EMT program in TNBC cell lines [15]. Interestingly, Lock et al. [28] demonstrated that the CA IX inhibitor U-104 (synonym of SLC-0111), depleting CSCs from breast cancer xenografts, enhanced the effect of paclitaxel in delaying tumor growth and reducing spontaneous metastasis in vivo. When the same compound was used in combination with TMZ, the standard of care chemotherapy in glioblastoma (GBM), it reduced brain tumor-initiating cells (BTIC) enrichment from xenograft-derived GBM cells treated with TMZ and tumor recurrence in GBM patient-derived xenografts [12]. It is well-known that the hypoxic/acidic TME reduces normal immune function. It has been reported that SLC-0111 by reducing extracellular acidification, augmented the effects of immune-checkpoint inhibitors anti–PD-1 and anti–CTLA-4 on enhancement of Th1 response and reduction of melanoma tumor growth and breast cancer metastasis [13]. Here, we demonstrate that hypoxic microenvironment causes resistance to Cis-Pt treatment and enhances CA IX/XII expression levels in HNSCC cells and report high expression levels of these proteins in a subset of HNSCC patients and not in normal human tissues through an in silico analysis. For the first time, we evaluated the possibility to increase the effects of Cis-Pt on HNSCC cell lines grown under hypoxic conditions inhibiting CA IX/XII with specific inhibitor SLC-0111. Our data highlight that combinatorial treatment is more effective than single chemotherapy in inhibiting tumor cell growth and invasiveness as well as in reducing number, size and spread in Matrigel of spheroids including the expression levels of their stemness markers Nanog and Sox-2. Furthermore, we demonstrate that SLC-0111 is able to potentiate Cis-Pt effect on altered signaling pathways involved in cancer progression such as STAT-3, AKT and ERK decreasing the expression levels of the phosphorylated/activated forms of these proteins. In addition, we report a greater inhibition of EMT program after combinatorial treatment respect to therapy with the single drugs as shown by the decrease of expression levels of mesenchymal markers N-cadherin and β-catenin as well as by the enhancement of already high levels of the epithelial marker E-cadherin. Interestingly, McDonald et al. showed that the administration of SLC-0111 in combination with gemcitabine in KRAS mutant pancreatic cancer animal model increased tumor cell death with concomitant inhibition of tumor growth and metastasis [14]. In accordance with this study, we demonstrate that the CA IX/XII inhibition improved the therapeutic efficacy of Cis-Pt in HNSCC xenografts increasing tumor cell death as shown by in vivo molecular imaging with NIR-Annexin V and by analysis of cleaved-form of caspase-3 and PARP expression levels from ex vivo tumors. Further, confirming the in vitro findings showing the ability of SLC-0111 to increase Cis-Pt inhibition of HNSCC cells and spheroid invasiveness, preliminary studies, performed in a highly invasive HNSCC orthotopic mouse model using NIR-imaging with ProSense-750, showed a greater reduction of metastatic spread after combinatorial treatment.

## Conclusions

The tumor hypoxic microenvironment affects the efficacy of chemotherapy, radiotherapy and immunotherapy in HNSCC. In this study, we identified the hypoxia-induced CA IX/XII as a node shared in therapy resistance and metastasis mechanisms in HNSCC. Furthermore, our results showing the ability of the specific CA IX/XII inhibitor SLC-0111 in sensitizing HNSCC cells and animal models to Cis-Pt in terms of reduced tumor growth and dissemination, highlight the possibility to use it as an integrated therapeutic approach to combat metastatic progression and overcome therapy resistance.

## Supplementary Information

**Additional file 1.**

## Data Availability

All data analyzed during this study are included in this manuscript.

## Abbreviations

ATCC: American Type Culture Collection
AKT: Protein Kinase B
ANOVA: One-way analysis of variance
BTIC: Brain tumor-initiating cells
CA IX/XII: Carbonic anhydrase isoenzymes IX and XII
Cis-Pt: Cisplatin
DMEM: Dulbecco Minimum Essential Medium
DPBS: Dulbecco’s phosphate-buffered saline
ERK1/2: Extracellular-signal regulated kinase 1/2
EMT: Epithelial-mesenchymal transition
FBS: Fetal bovine serum
FRI: Fluorescence Reflectance
FMT: Fluorescent Molecular tomography
GBM: Glioblastoma
HIF-1α: Hypoxia-Inducible Factor 1-alpha
HNSCC: Head and neck squamous carcinomas
HPV +: Human papillomavirus
OPSCC: Oropharyngeal carcinomas
OSCC: Oral carcinomas
MMP-2: Metalloproteinase-2
MTS: 3-(4, 5-Dimethylthiazol-2yl)-5-(3-carboxymethoxy-phenyl)-2-(4-sulfophenyl)-2H tetrazolium
NIR: Near-Infrared Reflectance
RM-ANOVA: Model analysis of variance for repeated measures
SDS-PAGE: Sodium Dodecyl Sulphate–PolyAcrylamide Gel Electrophoresis
STAT3: Signal Transducer And Activator Of Transcription 3
TMZ: Temozolamide
Imaging MFI: Mean fluorescence intensity
PI: Propidium iodide
PR: RPMI-1640: Roswell Park Memorial Institute-1640 medium
RT: Room temperature
TME: Hypoxic tumor microenvironment
