# Supplementary material for: Inhibition of carbonic anhydrases IX/XII by SLC-0111 boosts cisplatin effects in hampering head and neck squamous carcinoma cell growth and invasion
Source: J Exp Clin Cancer Res. 2022 Apr 2;41:122. doi: 10.1186/s13046-022-02345-x (PMC8976345; doi:10.1186/s13046-022-02345-x)
Supplement: Supplementary file 1 — Additional file 1. [file 13046_2022_2345_MOESM1_ESM.docx]

**Supplementary Information**

**SLC-0111 increases Cis-Pt effect on apoptosis in HNSCC cells**

**(A and C)** FaDu and SCC-011 cells grown under hypoxic conditions (1% O_2_) and treated with Cis-Pt (1 μM), SLC-0111 (100 μM) and combination of the two drugs were stained with Annexin V/PI and subjected to flow cytometry analysis. Values are shown relative to untreated cells, arbitrarily set to 1 (n=3). Bars depict mean ±SD of three independent experiments (** p<0.001; * p<0.01). **(B and D)** Lysates obtained from FaDu and SCC-011 cells treated as above described were immunoblotted with antibodies anti pro-caspase-3/cleaved-caspase-3 and PARP/cleaved-PARP. Equal loading was confirmed by immunoblot with anti-actin antibody. The graphs display the relative quantities of protein expression levels.

**
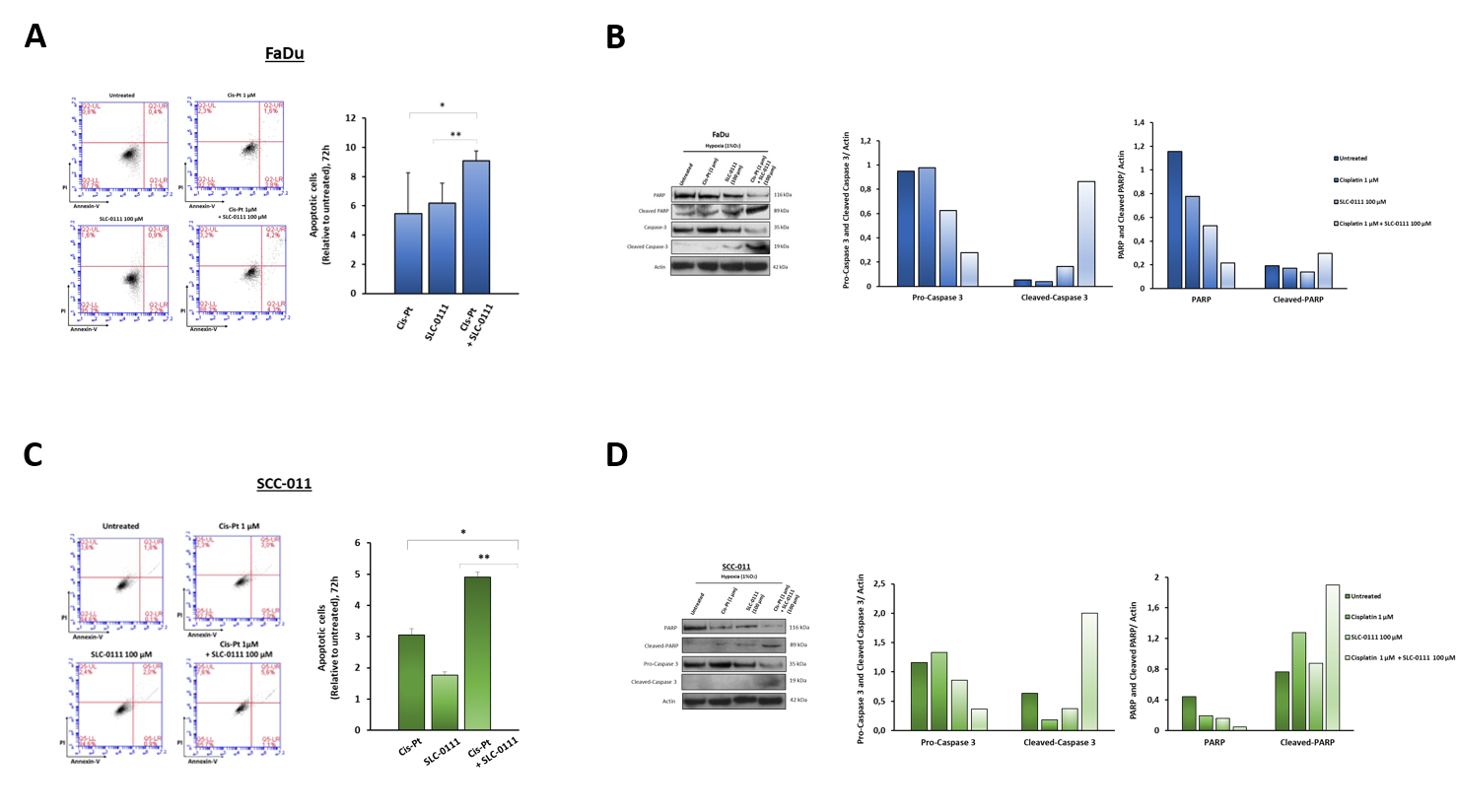
**

**Figure S1**

**The combination of SLC-0111 and Cis-Pt drastically reduces MMP-2 levels in FaDu xenografts.** Lysates from recovered FaDu xenografts, treated with vehicle, SLC-0111 (100 mg/kg per os administration, via oral gavage) or Cis-Pt alone (3 mg/kg per i.p. injection), or the two drugs in combination, were immunoblotted with antibody anti MMP-2. Equal loading was confirmed by immunoblot with anti-Actin antibody.


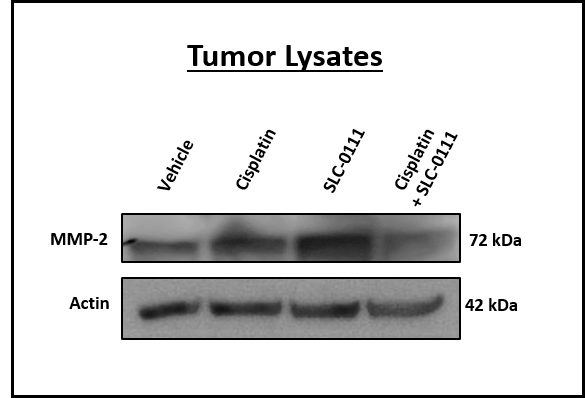


**Figure S2**
